# Predictive biomarkers for the progression of ocular complications in chronic Stevens-Johnson syndrome and toxic Eeidermal necrolysis

**DOI:** 10.1038/s41598-020-76064-8

**Published:** 2020-11-03

**Authors:** Yamato Yoshikawa, Mayumi Ueta, Hiromi Nishigaki, Shigeru Kinoshita, Tsunehiko Ikeda, Chie Sotozono

**Affiliations:** 1 Department of Ophthalmology, Osaka Medical College, Takatsuki-City, Osaka Japan; 2 Department of Ophthalmology, Kyoto Prefectural University of Medicine, Kyoto, Japan; 3 Department of Frontier Medical Science and Technology for Ophthalmology, Kyoto Prefectural University of Medicine, 465 Kajii-cho, Hirokoji-agaru, Kawaramachi-dori, Kamigyo-ku, Kyoto, 602-0841 Japan

**Keywords:** Biomarkers, Diseases, Medical research

## Abstract

This study aimed to clarify predictive biomarkers of mild and severe ocular complications of Stevens-Johnson syndrome (SJS) and toxic epidermal necrolysis (TEN) by examining the cytokines in tears. In 121 chronic-phase SJS/TEN eyes, cytokines in tear samples collected using Schirmer test strips were measured, and ocular sequelae severity was evaluated using an Ocular Surface Grading Score (OSGS) involving 7 components (conjunctivalization, neovascularization, opacification, keratinization, symblepharon, and upper/lower conjunctival-sac shortening), with findings categorized into grades 0–3 (maximum total OSGS: 21). Changes in cytokines between the mild and severe groups (mild: total OSGS of 10 or less, severe: total OSGS of 11 or more), and changes between SJS/TEN cases with and without each of the 7 components, were compared. In the severe group, there was significant upregulation of interleukin (IL)-8 (*P* < 0.01) and Granzyme B (GrzB) (*P* < 0.05). IL-8 was significantly upregulated in eyes with conjunctivalization, neovascularization, or opacification, GrzB was upregulated in eyes with keratinization, interferon-γ-inducible protein 10 (IP-10) was downregulated in eyes with conjunctivalization or neovascularization, and IL-1α was upregulated in eyes with opacification (all: *P* < 0.05). IL-8 and IP-10 was involved in conjunctivalization and neovascularization, while GrzB was involved in keratinization. IL-8 and GrzB in tears may reflect SJS/TEN-related ocular sequelae severity.

## Introduction

Stevens-Johnson syndrome (SJS) is an acute inflammatory disease that causes necrosis of the skin and mucous membranes. In SJS patients with extensive skin detachment and a poor prognosis, the disorder is termed toxic epidermal necrolysis (TEN). Symptoms of SJS and TEN include high fever and general malaise, erythema, erosion, blisters that frequently occur over the entire body, including the mouth, oral cavity, eyes, vulva, etc.^1–6^, which can ultimately lead to death due to concomitant severe complications such as sepsis, respiratory insufficiency, and multiple organ failure.

In the acute phase of SJS/TEN, severe ocular complications (SOC) reportedly occur in 50% of the patients^7–12^, and in the chronic phase, acute ocular surface damage causes severe scarring and symblepharon. In SJS/TEN cases, the SOC result in persistent visual loss and ocular discomfort, thus greatly affecting the patient’s quality of life (QOL).

In previous studies, we investigated the cytokines in tears of SJS/TEN cases with SOC, and found that in the acute phase, interleukin (IL)-6, IL-8, and monocyte chemoattractant protein-1 (MCP-1) were dramatically increased^13^, while in the chronic phase, there was a significant downregulation of interferon-γ-induced protein 10 (IP-10), as well as upregulation of IL-6, IL-8, eotaxin, and macrophage inflammatory protein-1 beta (MIP-1β) compared with the tears of normal control subjects^14^.

However, it remains unknown as to whether or not the inflammatory cytokines in tears differ depending on their severity of scarring of the ocular surface.

Recent studies have reported that SJS/TEN-related SOC progress in the chronic phase^15,16^, however, the biomarkers to predict the progression have yet to be elucidated. Moreover, the management of those SOC differs depending on each medical facility, so it is important to elucidate the specific predictive biomarkers to help guide treatment. In this study, we examined the differences in inflammatory cytokines in tears between mild and severe phenotypes of the SOC of SJS/TEN in order to elucidate the biomarkers.

## Results

**Figure 1:**
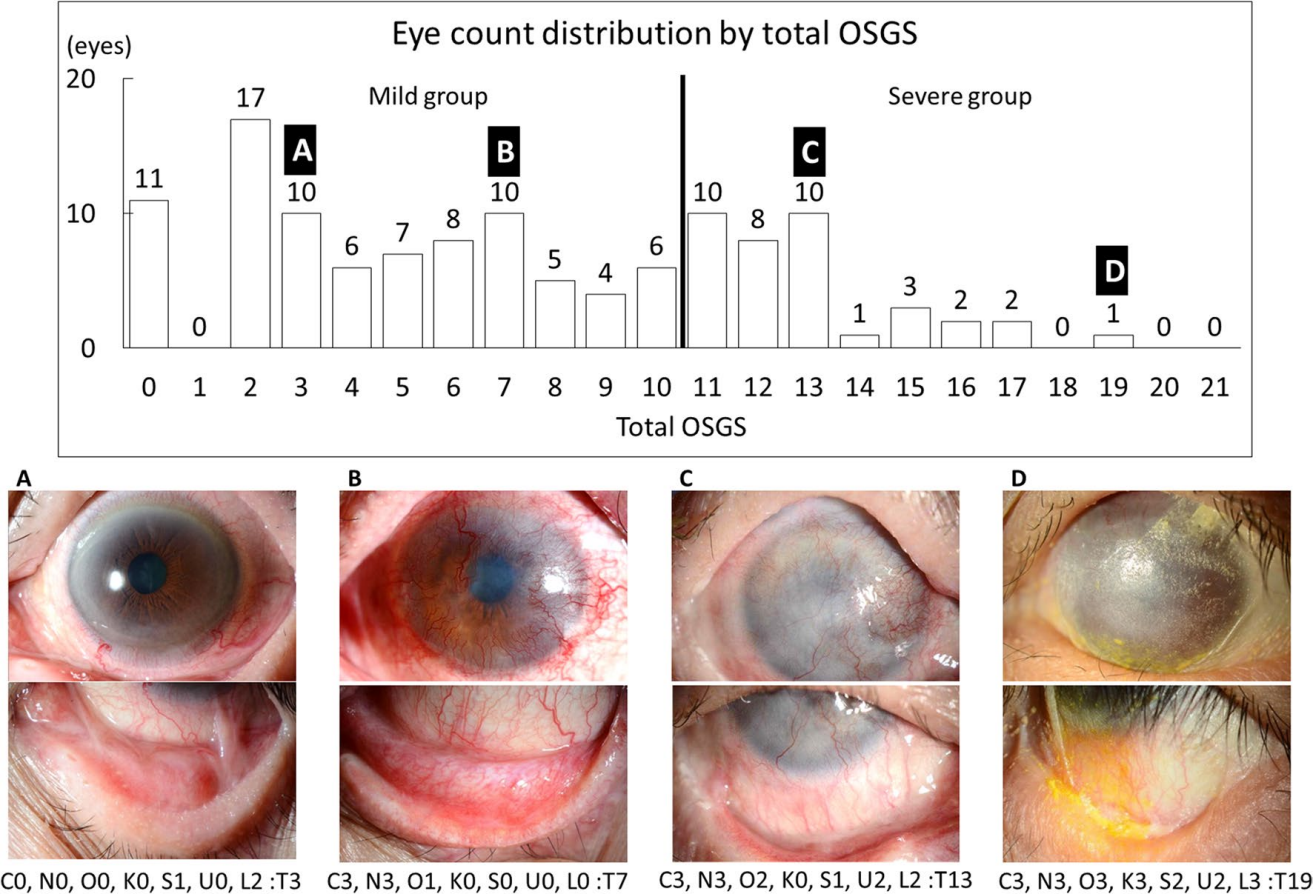
Eye count distribution by total Ocular Surface Grading Score (OSGS) and images of four representative Stevens-Johnson syndrome (SJS) or toxic epidermal necrolysis (TEN) patients. C, conjunctivalization; N, neovascularization; O, opacification; K, keratinization; S, symblepharon; U, upper conjunctival sac shortening; L, lower conjunctival sac shortening; T, total OSGS.

In this study, we compared the cytokines among the ‘mild group', ‘severe group', and ‘normal control group' tear samples. The distribution by total Ocular Surface Grading Score (OSGS) of the number of eyes in each group is outlined in Fig. 1. There were 84 eyes in the mild group and 37 eyes the severe group, and images of representative cases are shown in Fig. 1A–D.

### Comparison of cytokines between the mild and severe groups

**Figure 2:**
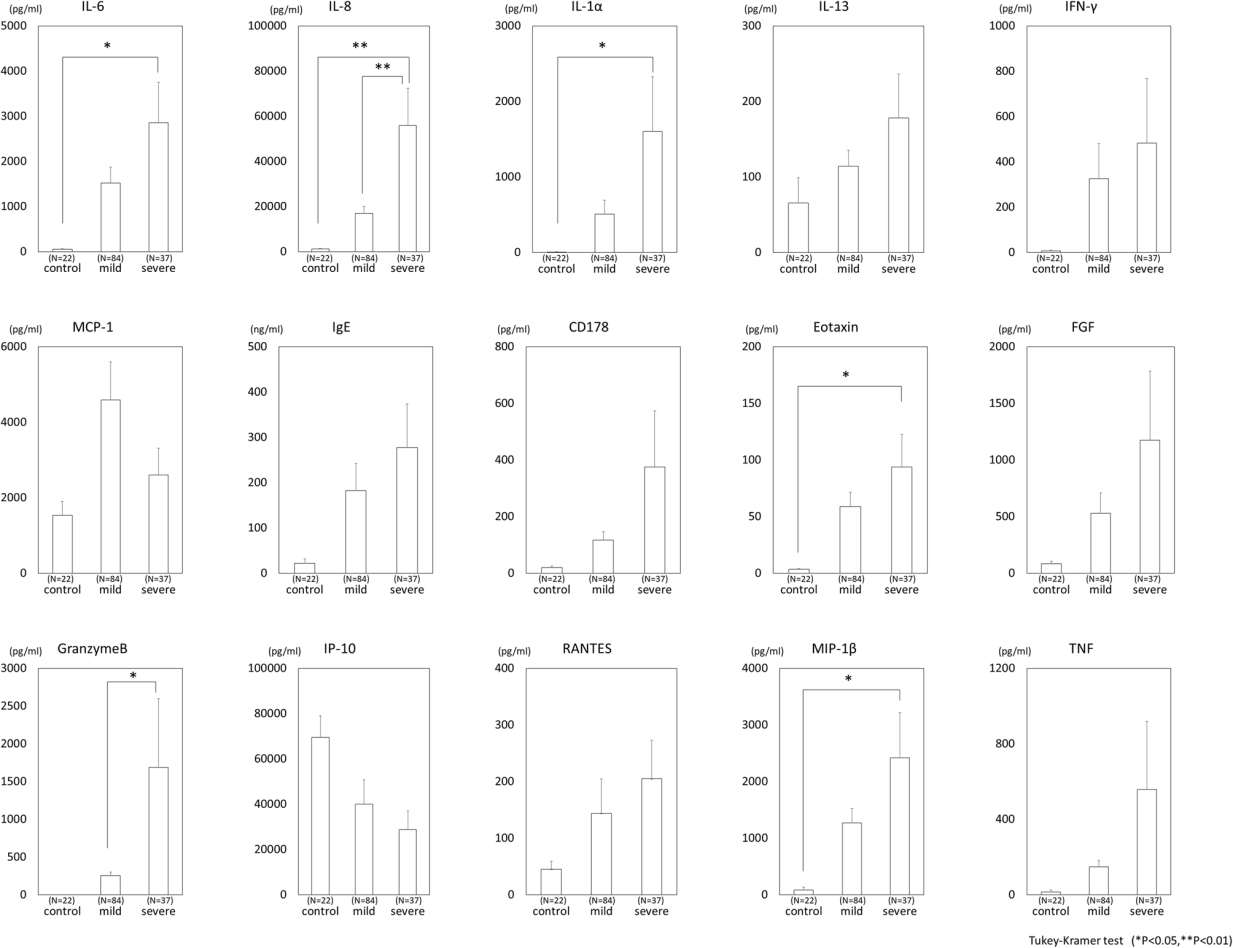
Comparison of cytokines and total immunoglobulin E (IgE) levels in tears from mild and severe cases of SJS/TEN patients with severe ocular complications and those from healthy control subjects. FGF, fibroblast growth factor; IFN-γ, interferon gamma; IgE, immunoglobulin E; IL, interleukin; IP-10, interferon-γ-inducible protein 10; MCP-1, monocyte chemoattractant protein-1; MIP-1β, macrophage inflammatory protein-1 beta; RANTES, regulated on activation, normal T cell expressed and secreted; TNF, tumor necrosis factor.

Compared to the mild group tears, the severe group tears had significant upregulation of IL-8 (*P* < 0.01) and Granzyme B (GrzB) (*P* < 0.05) (Fig. 2). This finding suggested that focusing on IL-8 and GrzB might be the predictive biomarkers for the progression of SOC in SJS/TEN. Although there was no significant difference found in regard to the other cytokines, many cytokines tended to increase in the severe group.

### Relationship between cytokines in tears and components of the ocular surface

**Figure 3:**
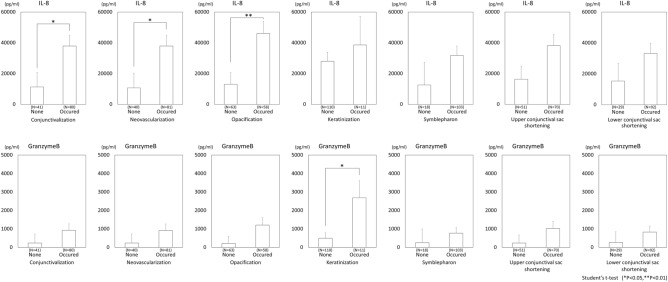
Comparison of IL-8 and Granzyme B between SJS/TEN cases with and without each of the 7 ocular-surface components.

We examined the difference in IL-8 and GrzB between the SJS/TEN cases with and without each of the 7 components, and the results are shown in Fig. 3. In the SJS/TEN cases with conjunctivalization, neovascularization, or opacification, IL-8 was significantly upregulated (*P* < 0.05, *P* < 0.05, and *P* < 0.01, respectively). In the SJS/TEN cases with keratinization, GrzB was significantly upregulated (*P* < 0.05). Representative cases without conjunctivalization (C), neovascularization (N), and opacification (O) are shown in Fig. 1A (C: 0, N: 0, and O: 0). Representative cases with conjunctivalization, neovascularization, and opacification are shown in Fig. 1B (C: 3, N: 3, and O: 1)*,* C (C: 3, N: 3, and O: 2)*,* and D (C: 3, N: 3, and O: 3). Representative cases without keratinization (K) are shown in Fig. 1A (K: 0)*,* B (K: 0)*,* and C (K: 0). Representative cases with keratinization (K) are shown in Fig. 1D (K: 3)*.*

**Figure 4:**
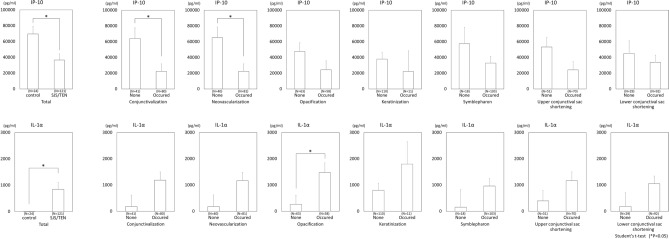
Comparison between the normal control subjects and all SJS/TEN cases, and IP-10 and IL-1α between SJS/TEN cases with and without each of the 7 ocular-surface components.

It was possible that many of the cytokines measured in this study were not significantly different between the mild and severe cases due to the interplay of multiple components of the ocular surface. Our findings revealed a relationship between IL-8 and GrzB and the specific components of ocular surface. Hence, we then compared other cytokines between SJS/TEN cases with and without each of the 7 components. Upon investigation, IP-10 and IL-1α showed a significant difference between SJS/TEN cases with and without some components (Fig. 4). IP-10 was significantly downregulated In the SJS/TEN cases with conjunctivalization or neovascularization (*P* < 0.05, *P* < 0.05, respectively). Moreover, IL-1α was significantly upregulated In the SJS/TEN cases with opacification (*P* < 0.05). However, no significant difference was observed in regard to the other cytokines.

## Discussion

In this study, our findings revealed that IL-8 and GrzB were upregulated in the tears of severe SJS/TEN cases compared to the tears of mild SJS/TEN cases. When examined for association with each component of ocular surface, our findings revealed a relationship between cytokines IL-8, GrzB, IP-10, and IL-1α and specific components of ocular surface.

IL-8 was significantly upregulated in the SJS/TEN cases with conjunctivalization, neovascularization, or opacification. In our previously study investigating the progression of 7 components of the ocular surface in chronic SJS/TEN cases, our findings revealed that there is a significant correlation between conjunctivalization, neovascularization, and opacification^15^. It has previously been reported that IL-8 promotes corneal neovascularization in mouse models^17,18^. Therefore, it is quite reasonable that IL-8 was found to be upregulated in these three specific disease components. Our findings possibly suggest that the suppression of IL-8 can suppress the progression of conjunctivalization and neovascularization.

Compared to the mild group tears, we found that GrzB was upregulated in the severe group tears. However, GrzB was not detected in any of the normal control group tear samples. In the SJS/TEN cases, GrzB was detected in 70.2% (85 of 121 eyes). Interestingly, the upregulation of GrzB was found to be significantly higher in SJS/TEN cases with keratinization. It should be noted that GrzB is a mediator that reportedly works with perforin in the apoptotic pathway in cases of SJS/TEN^5,19–21^. Although the role of GrzB on the ocular surface has yet to be fully elucidated, our findings might possibly indicate that keratinization can be caused by the upregulation of GrzB. Thus, the inhibition of GrzB might help contribute to the suppression of keratinization, and might be an effective treatment to reduce the progression of SOC in chronic SJS/TEN cases.

IP-10 was significantly downregulated in all SJS/TEN cases compared to the normal control subjects, similar to the findings in previous reports^14,22^. Moreover, IP-10 was significantly downregulated in SJS/TEN cases with conjunctivalization and neovascularization. As stated above, conjunctivalization is strongly correlated with neovascularization^15^. It was previously reported in a corneal-neovascularization mouse-model study that IP-10 acts as an inhibitor of neovascularization^23^. Hence, it is possible that the downregulation of IP-10 contributes to the progression of conjunctivalization and neovascularization in SJS/TEN cases, and that upregulation of IP-10 might suppress the progression of conjunctivalization and neovascularization. Another studies noted upregulation of IP-10 in eyes with evaporative DED and downregulation of IP-10 in eyes with chronic GVHD^24,25^. We have found that in eyes with SJS/TEN, IP-10 was downregulated as same as GVHD^14^.

IL-1α was significantly upregulated in the SJS/TEN cases with opacification. IL-1α reportedly increases during the wound healing process of corneal stromal scarring^26^. Similarly, IL-1α may have been upregulated in SJS/TEN cases with opacification.

This study might have a limitation due to the cross sectional study. In the future, prospective studies with multiple cytokine measurements over time are needed.

In conclusion, the findings of this study showed that IL-8 in tears might be a biomarker of conjunctivalization and neovascularization, and similarly, GrzB might be a biomarker of keratinization. Moreover, our findings revealed that IP-10 might be a biomarker for the suppression of conjunctivalization and neovascularization. Thus, treatment aimed at maintaining lower levels of IL-8 and GrzB and higher levels of IP-10 may suppress the progression of SOC in chronic-phase SJS/TEN cases. It should be noted that to the best of our knowledge, this is the first report to illustrate the relationship between tear cytokines and the pathological components of the ocular surface in chronic-stage SJS/TEN cases.

## Methods

### Subjects

The protocol of this study was approved by the Ethics Review Board of Kyoto Prefectural University of Medicine, Kyoto, Japan, and in accordance with the tenets set forth in the Declaration of Helsinki, written informed consent was obtained from all subjects prior to their involvement in the study. Our investigation involved the use of tear samples obtained from 121 eyes of SJS/TEN patients in the chronic stage (i.e., at more than 1 year from disease onset) who were seen between 2016 and 2019 in the Department of Ophthalmology Outpatient Clinic at the Kyoto Prefectural University of Medicine. The average duration of chronicity from onset was 22.9 ± 17.3 years. To meet the inclusion guidelines, all subjects were SJS/TEN patients with SOC. For a control, we also obtained tear samples from 24 eyes of healthy volunteer subjects with no history of ocular disease.

### OSGS

The SJS/TEN patients were divided into two groups, i.e., ‘mild' and ‘severe', according to our previously reported OSGS^27,28^. Briefly, in accordance with our modified OSGS^15^, the following 7 ocular-surface components were examined in all enrolled SJS/TEN patients: (1) conjunctivalization, (2) neovascularization, (3) opacification, (4) keratinization, (5) symblepharon, and (6) upper and (7) lower conjunctival sac shortening. Each component was graded on a scale from 0 to 3, depending on the severity of involvement. The OSGS results were then combined to produce a total OSGS ranging from 0 to 21, with 21 representing the most severely affected eyes. All patients were divided into 2 groups by total OSGS, with those with a score of 10 or less being considered as the 'mild group' and those with a score of 11 or more being considered as the ‘severe group'.

### Cytokines

In the SJS patients with ocular complications, we measured the amount of tear cytokines [i.e., IL-6, IL-8, IL-1α, IL-13, interferon-γ, MCP-1, total immunoglobulin E (IgE), CD178, eotaxin, fibroblast growth factor (FGF), GrzB, IP-10, regulated on activation, normal T cell expressed and secreted (RANTES), MIP-1β, and tumor necrosis factor (TNF)].

Tear samples were collected on Schirmer Tear Production Measuring Strips (Showa Yakuhin Kako Co., Ltd., Tokyo, Japan) according to our previously reported method^14^. They were immersed in 100 µL Tris-buffered saline, we measured the titre of the above-listed cytokines in 50 µL of TBST containing the tears. The tear volume on the Schirmer test strips was calculated at 1 µL intervals using a standard curve obtained from 0 to 25 µL of distilled water. The concentration of cytokines in the tear samples was measured with the BD CBA Flex Sets and BD human soluble protein master buffer kits (BD Biosciences) .Concentrations were calculated with FCAP Array Software version 3.0 (BD Biosciences)^14^.

### Statistical analysis

Data were expressed as mean and individual values, using JMP PRO version 14 (SAS Institute, Inc., Cary, NC) software. Comparisons between the three groups were evaluated using the Tukey–Kramer test, and comparisons between the mild and severe SJS/TEN groups were evaluated using Student's t-test. In all tests, a *P*-value of < 0.05 was considered statistically significant.
